# Go/no-go task performance of Japanese children: Differences by sex, grade, and lifestyle habits

**DOI:** 10.3389/fpubh.2022.883532

**Published:** 2022-08-22

**Authors:** Akiko Shikano, Shingo Noi

**Affiliations:** Research Institute for Health and Sport Science, Nippon Sport Science University, Tokyo, Japan

**Keywords:** sleep, screen time, physical activity, cognitive function, commission error, omission error

## Abstract

**Background:**

Japanese children face critical psychological challenges that urgently need to be addressed.

**Objective:**

This study aimed to clarify performance differences in go/no-go tasks among Japanese elementary and junior high students by sex and grade and comprehensively investigate the relationship between children's lifestyle habits and performance.

**Methods:**

In total, 4,482 (2,289 males, 2,193 females) 1st grade elementary to 3rd grade junior high students (6–15 years old) participated. We conducted a survey and the go/no-go experiments in the participating schools on weekday mornings from November 2017 to February 2020. We collected data on the number of errors in the go/no-go tasks in response to visual stimuli (commission errors in the no-go tasks; omission errors in the go tasks); and on lifestyle habits (i.e., sleep, screen time, and physical activity) using questionnaires.

**Results:**

For the commission errors, the results demonstrated differences by sex and grade; for the omission errors, differences were only observed by grade. Additionally, we analysed the relationship between both types of errors and sex, grade, sleep conditions, screen time, and physical activity using binomial logistic regression analysis. Commission errors were significantly related to sex and grade whereas omission errors were related to grade, bedtime, screen time, and physical activity.

**Conclusions:**

Our results highlighted that children's cognitive functions are related to their lifestyle habits (i.e., sleep conditions, screen time, and physical activity) in addition to sex and grade.

## Introduction

The UNICEF Innocenti Report Card 16 (1), published in September 2020, uses a variety of comparable data from 38 Organisation for Economic Co-operation and Development or European Union member countries to rank the well-being of children in each country in three domains: good mental well-being, good physical health, and skills for life. The results showed that Japanese children ranked first in “good physical health”, but extremely low in “good mental well-being”, at 37th place. Suicide, bullying, violence, and long-term absenteeism have been identified as social problems among Japanese children. The United Nations Committee on the Rights of the Child has expressed its concerns in the Concluding Observations on the Combined Fourth and Fifth Periodic Reports concerning Japan in sections on “Research the root causes for suicide among children, implement preventive measures”, in paragraph 20 (b); and on “Implement effective measures against bullying”, in paragraph 39 (a) (2). Furthermore, paragraph 20 (a) goes so far as to identify the need to “Take measures to ensure that children enjoy their childhood, without their childhood and development being harmed by the competitive nature of society.” Additionally, since the 1990s, problematic events such as a “breakdown in classroom discipline” and “sudden anger outbursts” have also been reported in Japanese schools (3). These problems suggest that Japanese children face critical psychological challenges that urgently need to be addressed.

Many studies suggest that the physical basis of the mind lies in the brain; consequently, it should be possible to confirm some of the features of the mind by studying brain functions. Thus, many studies on brain structure and function have been conducted using non-invasive measurements of brain activity (e.g., magnetic resonance imaging, electroencephalography, and near-infrared optical topography), which have been regarded as indicating physical reactions of the mind. Through these measurements, it is possible to observe brain function in real-time in terms of perceptions, movements, and cognition. Such scientific advancements have allowed for a deeper understanding of children's development and disabilities. However, although such measurements are non-invasive, their implementation is extremely difficult in the context of childcare and education because they require expensive equipment and specialised technology. Due to these limitations, observational approaches (e.g., cognitive function tests) to measure children's actions and activities, regarded as the output of their brain activity, may be more useful and easily applied. Specifically, many studies have used go/no-go tasks as one type of observational approach.

Casey et al. (4) investigated the development of cognitive function in children aged 4- to 18-years-old, and reported a decrease in reaction time between the ages of 4 and 12 years; likewise, Durston et al. (5) used go/no-go tasks as an index to analyse neurological deficits related to cognitive function in children with attention deficit hyperactivity disorder (ADHD) aged 6 to 11 years; many other studies share similarities with the research mentioned above [e.g., (6, 7)]. One of the positive outcomes of such studies relates to the applicability of their findings; they may serve not only as theoretical support to field measurements of go/no-go tasks for children but also provide effective and reliable techniques that are useful when researchers want to develop measures to understand psychological disabilities. This method of measurement has also been long used intensively in Japan (3, 8, 9).

Furthermore, many studies have found a relationship between brain function and lifestyle habits, such as between sleep and physical activity (PA); for example, Touchette et al. (10) examined sleep duration patterns, behavioural characteristics, and cognitive function in children from school entry to elementary school. These authors reported a relation between short sleep durations and hyperactivity-impulsivity and between short sleep durations and children's cognitive test results. Other studies have found that sleep deprivation impairs cognitive function [e.g., (11)], and that taking a nap improves executive function tasks and affects prefrontal activity (12). Regarding PA, transient moderate-intensity exercise reportedly accelerates response time in visual choice reaction tasks (13), and the more PA a person undertakes, the faster the reaction to flanker tasks (14). Furthermore, children with low inhibitory control capabilities can be expected to experience greater effects from undergoing PA (15). Recently, researchers have raised concerns regarding the effects of Internet use on brain function. Horowitz-Kraus and Hutton (16) reported that the functional connectivity between the visual word form area and the left-sided language, visual, and cognitive control region of the brain increases with reading but decreases with longer periods of media use. Moreover, another study reported that Internet addicts have widespread white matter abnormalities (17). Given these findings, concerns have arisen in Japan regarding the possible negative effects of Internet use on children's cognitive function, as their use of the Internet is increasing (18).

However, these studies examined the relationship between specific lifestyle habits and cognitive functions using univariate analysis; hence, they did not investigate the possible effects of a combination of the children's different lifestyle habits. In everyday life, children's sleep, physical activity, screen time, and other aspects of their lives are all interrelated (19). This consideration led the WHO to develop 24-h behavioural guidelines that include recommendations on screen time and sleep duration, in addition to physical activity, to ensure the greatest health benefit (20). Therefore, this study aimed to clarify performance differences regarding go/no-go tasks among Japanese elementary and junior high students by sex and grade. Specifically, we intended to comprehensively investigate the relationship between children's lifestyle habits (e.g., sleep, screen time, and PA) and their performance.

## Materials and methods

### Ethics approval and consent to participate

This study was conducted with the approval of the Ethics Review Committee for experiments on humans, Nippon Sport Science University (Approval No. 017-H092). Before participation, all potential participants and their guardians were provided with a written explanation concerning the purpose and content of the study, and all participants provided written informed consent. Measures were taken to ensure the anonymity of all collected data.

### Participants

In total, there were 4,482 (2,289 males, 2,193 females) participants, spanning from 1st graders in elementary school to 3rd graders in junior high school (age range: 6–15 years old). Participants were recruited through snowball sampling from seven public elementary and three public junior high schools in eight Japanese cities (two urban and six suburban). No participants had any medical or psychological problems that could have affected study results.

### Measures and procedure

The study was conducted on weekday mornings from November 2017 to February 2020 on days when there were no special school events. We collected data on children's grasp motor responses to go/no-go tasks by visual stimulation and on their lifestyle habits (i.e., sleep, screen time, and PA) through a questionnaire.

#### Go/no-go task

**Figure 1:**
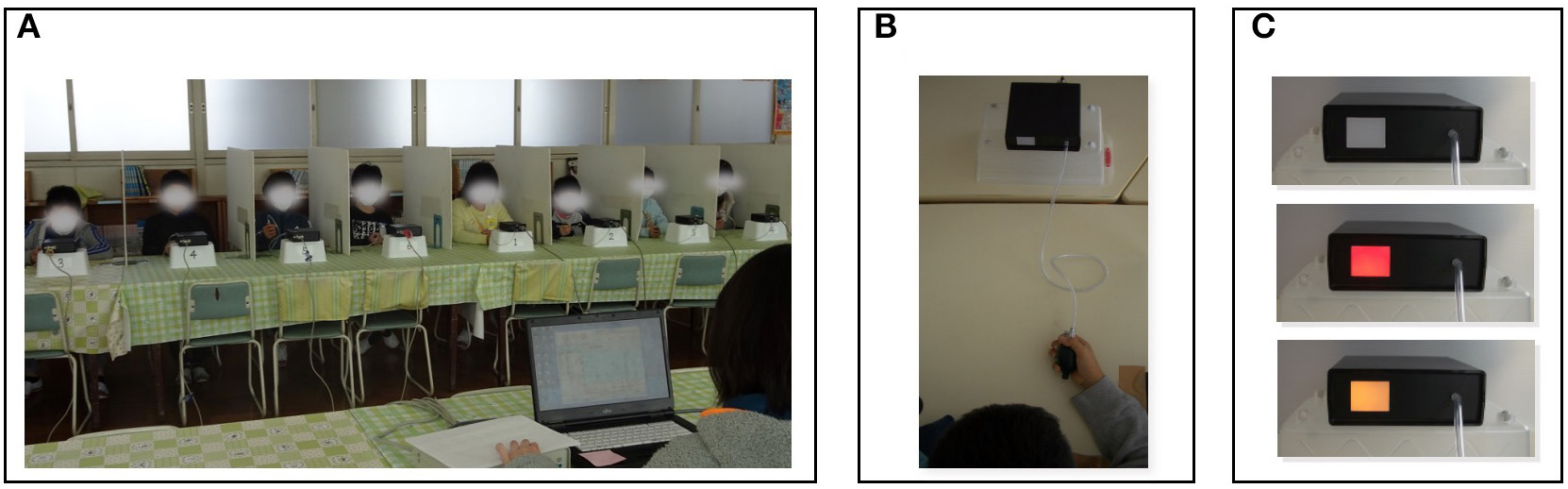
The go/no-go task in this study. The experiment was conducted in groups of up to 12 participants; **(A)** A stimulator was placed in front of each participant, and their responses were collected from grasping the rubber balls; **(B)** Go trials were a red light for elementary school children or a bright light for junior high school children; no-go trials were a yellow light for elementary school children or a dark light for junior high school children; **(C)** Both trials consisted of 11 phases, totalling 22 phases.

The present study was conducted following the go/no-go task methodology that has been previously used in Japan (3, 8, 9). Specifically, using a cerebral activity measurement program (made by Techno Muscat), data on go/no-go tasks were collected from groups of up to 12 individuals. The setting was a quiet classroom at each school, and there were no other persons present other than the participants and the investigators. Each participant sat on a chair and was separated from the next participant by a partition (Figure 1). The participants were instructed to use a rubber ball, held in their dominant hand, to respond to a visual stimulus (4 cm × 2.5 cm) provided by a light that was projected in front of a box (10.5 cm × 4.5 cm). The box was placed approximately 50 cm from and in front of the participant (metrics dictated by the investigator's rules). For elementary school students, the light stimulus referred to distinct colours (red and yellow lights); for junior high students, the stimulus referred to the distinction between light and darkness (170 nit and 30 nit). The task procedures were carried out in this order: first, the formation, and then the differentiation experiment (Figure 1), as described below.

In the formation experiment, the participants were instructed as follows: “From now on, the light in front of you will shine (for an elementary school student: “red”; for a junior high student: “bright”). When the light shines, grasp the rubber ball as quickly as possible. When the light goes off, release it”. They then practised this ten times. Immediately afterwards, visual stimuli (length: 0.5–1.5 s) were presented five times at random intervals (from 3 to 6 s).

In the differentiation experiment, the participants were instructed as follows: “There will be times when the light will be (for an elementary school student: “yellow”; for a junior high student: “dark”). When this happens, do not grasp the ball. As in the previous case, only grasp it as quickly as possible when you see the light become (for an elementary school student: “red”; for a junior high student: “bright”)”. They then practised this twice for each task (go task: 2 times; no-go task: 2 times). Immediately afterwards, visual stimuli (length: 0.5–1.5 s) were presented 22 times (11 trials for each task, go and no-go) at random intervals (from 3 to 6 s). The data from the differentiation experiment were then analysed.

Previous studies have used similar experimental procedures related to go/no-go tasks with at least 100 trials. However, this study used fewer trials (5 and 22 for the formation and differentiation experiments, respectively) because the experiment involved children in a childcare/education context (in which time is limited) and this number of trials has been shown to be efficient and useful in Shikano and Noi's (8) study.

#### Questionnaire

Children's lifestyle habit data were collected using a self-administered questionnaire. We utilised a collective survey method. The survey was conducted in a classroom other than the one used for the go/no-go tasks, but both procedures were always conducted on the same day. Owing to first and second-graders' (elementary school) need for longer times to answer the questionnaire and the low reliability of their answers, we did not conduct the survey with them. Hence, we collected questionnaire data from 3,217 (1,664 males, 1,553 females) participants.

Our questionnaire was based on the lifestyle survey conducted by the Committee for Surveillance of Health in School Children and Adolescents (18) and Noi and Shikano (21). The items included: bedtime on the day before the survey; wake-up time on the day of the survey; daily mobile phone, smartphone, tablet, and PC usage time (i.e., screen time); and the number of exercise sessions per week (hereinafter PA). Sleep duration was estimated based on the recorded bedtime and wake-up times.

#### Data analysis

In this study, we examined the following three points:

1. Participants' lifestyle habits, namely, bedtime, wake-up time, sleeping hours, screen time, and PA. These were described by means, standard deviations (*SD*), and range (lowest value-highest value). Following the aggregation method of two previous studies (18, 19), we calculated and differentiated these variables by sex and grade. Further, we also calculated the number and percentages of participants who did not engage in PA (0 times) and the median number times for those who did.
2. Differences in participants' number of errors in the differentiation experiment by sex and grade (commission errors regarding no-go tasks; omission errors with respect to go tasks). These were compared using non-repeating two-way analysis of variance. Additionally, we used the Bonferroni method to test the simple main effects when significant interactions were observed.
3. The relationship between lifestyle habits and commission and omission errors. After confirming the distribution of these errors, participants with less than the mean value of commission errors (3.3 times) were classified into a “low-value group”, and those with values equal to or higher than the mean were classified into a “high-value group”. A similar procedure was used for the omission errors. Those with 0 errors were classified into a “low-value group”, and those with 1 or more errors into a “high-value group”. After confirming the distribution of each answer regarding lifestyle habits, we provided similar classifications for the participants in relation to other variables. For bedtime and screen time, participants were classified using the mean and *SD*. Participants with a mean value that was < -0.5 *SD* were classified into a “mean < -0.5 *SD* group”; those with a mean that was −0.5 *SD* or more and < +0.5 *SD* were classified into a “mean ± 0.5 *SD* group”; and those with a mean that was +0.5 *SD* or more were classified into a “mean +0.5 *SD* or more group”. Regarding PA, there was a substantial number of participants who did not engage in any PA. Participants who engaged in PA 0 times were classified into a “no activity group”; those below the median (excluding those with 0 times) into a “median or below group”, and those above the median into a “above the median group”. After these classifications, we conducted a multivariate binomial logistic regression analysis (forced input method), with commission and omission errors as dependent variables (low-value group = 0, high-value group = 1), and lifestyle habits (bedtime, screen time, PA), sex, and grade as independent variables. Wake-up time and sleep duration were excluded owing to the possibility of between-variable multicollinearity.

As data on lifestyle habits were not collected from elementary school 1st and 2nd graders, data from elementary 3rd graders to junior high 3rd graders were analysed for the first and third points of the above examinations and data from elementary 1st graders to junior high 3rd graders were analysed for the second point of the above examination. We used IBM^®^ SPSS^®^ ver. 26 software for statistical analysis, with the statistical significance rate set at <5%.

## Results

### Participants' lifestyle habits

Table 1 illustrates the participants' bedtime, wake-up time, sleep duration, screen time, and PA data. Our results demonstrated that, for both male and female students, the general tendency was that as grades increased, sleep duration shortened because bedtime occurred later and wake-up time occurred earlier. For both male and female students, mean screen time ranged from 64.6 to 151.9 min per day, and it increased with grade. Furthermore, 620 participants (19.3%) did not engage in any PA per week. Among those who engaged in PA, the median number of PA sessions per week was 4 (2,597, 80.7%).

**Table 1:** Participants' bedtime, wake-up time, sleep duration, screen-time, and physical activity (Per Week).

		***n***	**Mean ±*SD***	**Range**	**Median**
**Bedtime (h:m** **±** **m)**
Male students	3rd−4th	619	21:49 ± 56	18:30–3:10	21:40
	5th−6th	659	22:15 ± 65	18:00–4:40	22:00
	JHS 1st−3rd	386	22:55 ± 80	19:00–3:30	23:00
Female students	3rd−4th	552	21:41 ± 51	18:30–1:00	21:30
	5th−6th	588	22:22 ± 63	18:30–5:00	22:15
	JHS 1st−3rd	413	23:04 ± 77	18:00–4:00	23:00
**Wake-up time (h:m** **±** **m)**
Male students	3rd−4th	619	6:40 ± 35	3:50–9:30	6:45
	5th−6th	659	6:39 ± 40	1:15–11:00	6:45
	JHS 1st−3rd	386	6:25 ± 44	3:00–10:00	6:25
Female students	3rd−4th	552	6:39 ± 32	4:05–12:00	6:39
	5th−6th	588	6:38 ± 33	3:00–8:04	6:45
	JHS 1st−3rd	413	6:24 ± 39	4:10–9:30	6:15
**Sleep duration (h**^**h**^ **m**^**m**^ **±** **m**^**m**^**)**
Male students	ES 3rd−4th	619	8^h^51^m^ ± 59^m^	4^h^40^m^-12^h^20^m^	9^h^00^m^
	ES 5th−6th	659	8^h^24^m^ ± 68^m^	2^h^03^m^-12^h^30^m^	8^h^30^m^
	JHS 1st−3rd	386	7^h^29^m^ ± 73^m^	2^h^30^m^-11^h^00^m^	7^h^35^m^
Female students	ES 3rd−4th	552	8^h^58^m^ ± 52^m^	5^h^50^m^-13^h^50^m^	9^h^00^m^
	ES 5th−6th	588	8^h^16^m^ ± 65^m^	1^h^00^m^-12^h^10^m^	8^h^25^m^
	JHS 1st−3rd	413	7^h^20^m^ ± 70^m^	2^h^50^m^-12^h^00^m^	7^h^30^m^
**Screen time (minutes per day)**
Male students	ES 3rd−4th	619	95.0 ± 135.5	0.0–1201.0	60.0
	ES 5th−6th	659	114.8 ± 145.3	0.0–1080.0	60.0
	JHS 1st−3rd	386	142.5 ± 115.5	0.0–660.0	120.0
Female students	ES 3rd−4th	552	64.6 ± 100.9	0.0–660.0	30.0
	ES 5th−6th	588	114.0 ± 140.5	0.0–1020.0	60.0
	JHS 1st−3rd	413	151.9 ± 131.2	0.0–780.0	120.0
**Number of physical activity (times per week)**
Male students	ES 3rd−4th	619	4.5 ± 3.8	0.0–35.0	5.0
	ES 5th−6th	659	3.6 ± 2.8	0.0–21.0	4.0
	JHS 1st−3rd	386	3.9 ± 2.8	0.0–12.0	5.0
Female students	ES 3rd−4th	552	3.5 ± 3.2	0.0–30.0	3.0
	ES 5th−6th	588	2.7 ± 2.5	0.0–28.0	3.0
	JHS 1st−3rd	413	2.8 ± 2.9	0.0–12.0	5.0

*ES, Elementary school; JHS, Junior high school. The median values of physical activity were calculated based on students who exercised at least once per week*.

### Difference in errors by sex and age

**Figure 2:**
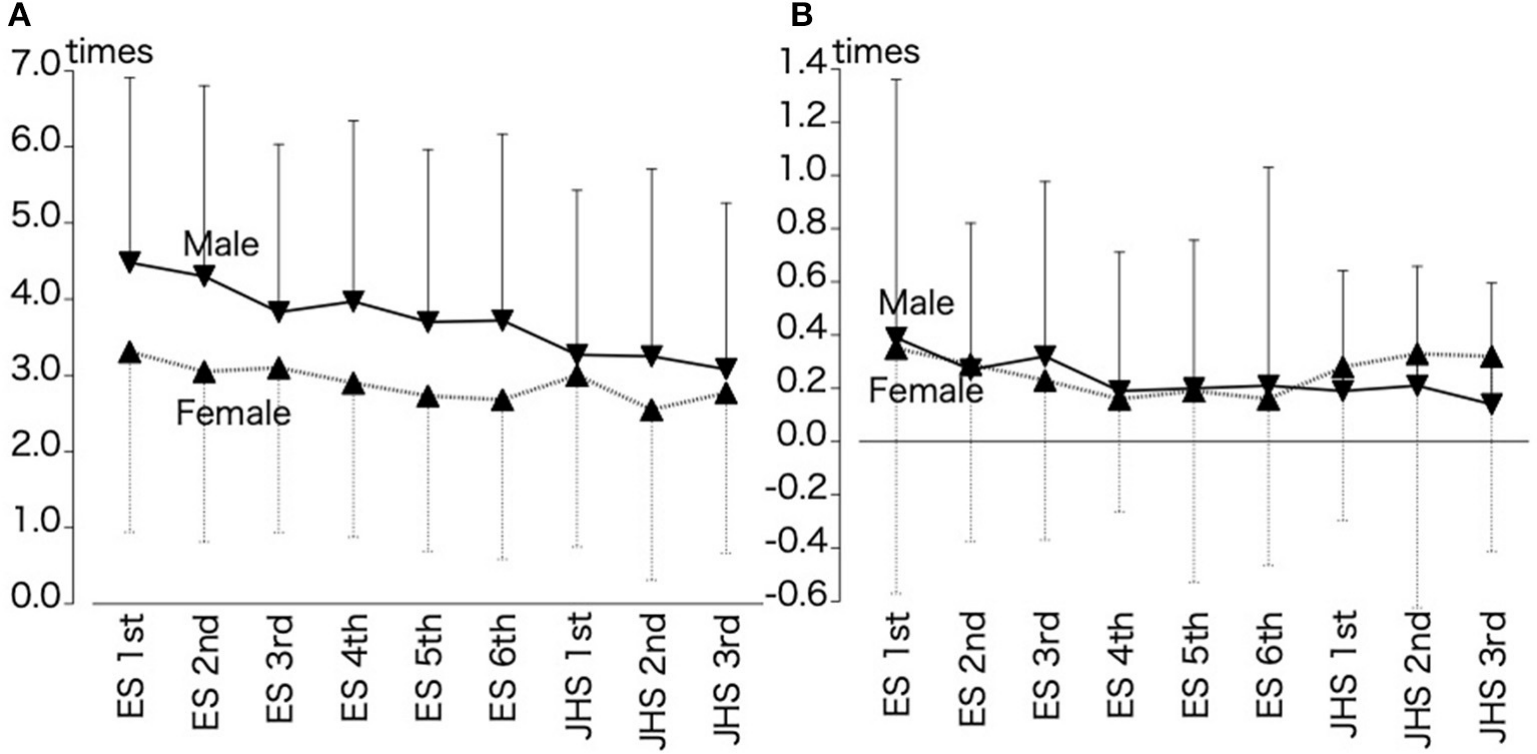
Cross-sectional changes in participants' commission **(A)** and omission errors **(B)** by grade and sex. ES, Elementary school; JHS, Junior high school. Mean + standard deviation. ▾ indicates male students (*n* = 2,289), ▴ indicates female students (*n* = 2,193).

Figure 2 illustrates the cross-sectional transition in participants' errors (commission and omission errors) by sex and grade, and Table 2 illustrates the differences in the number of errors (commission errors, omission errors) by sex and grade. Our results demonstrated a significant interaction between the sex and grade factors for commission errors [*F* (1, 8) = 2.320, *p* < 0.05]. Moreover, the simple main effect of sex was significant in grades, excluding junior high 1st and 3rd graders [*F* (1, 8) = 130.640, *p* < 0.05, male > female]. Additionally, the simple main effect of grade was significant for both male and female students [*F* (1, 8) = 9.615, *p* < 0.05, male: 1st elementary > 3rd/5th/6th elementary and 1st/2nd/3rd junior high; 2nd elementary > 5th/6th elementary and 1st/2nd/3rd junior high; 3rd/4th elementary > 3rd junior high; female: 1st elementary > 6th elementary/2nd junior high]. In contrast, no significant interaction was observed for omission errors; however, the simple main effect of grade (1st elementary > 4th/5th/6th elementary) was significant.

**Table 2:** Participants' commission and omission errors by sex and grade.

	**Factor**	**Degrees of freedom**	***F* value**	***p-*value**	**Partial eta-squared**
Commission errors	Sex	1	130.640	<0.001	0.028
	Grade	8	9.615	<0.001	0.017
	Sex × grade	8	2.320	0.018	0.004
Omission errors	Sex	1	0.813	0.367	0.000
	Grade	8	5.055	<0.001	0.009
	Sex × grade	8	1.504	0.150	0.003

*Data were analysed using a two-way ANOVA without repetition. This table reflects data collected from elementary 3rd graders to junior high 3rd graders (n = 3,217)*.

### Relationship between the number of errors and lifestyle habits

**Figure 3:**
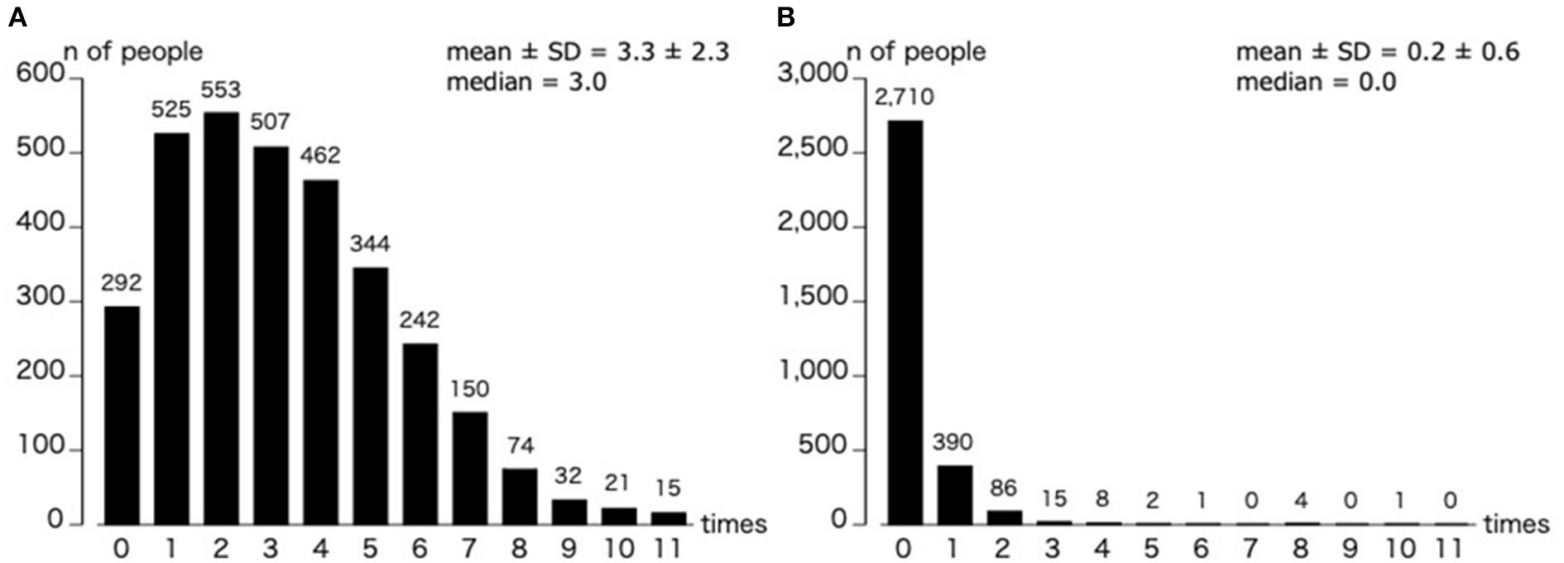
Distribution of commission errors **(A)** and omission errors **(B)** in the differentiation experiment. The results were analysed using the data from 3rd grade of elementary school to 3rd grade of junior high school. *n* = 3,217.

Figure 3 illustrates the number of errors for elementary 3rd graders to junior high 3rd graders. Our results demonstrated that the most common frequency of commission errors was 2. The distribution gradually decreased as the number of errors increased (error range: 0 to 11); and the mean value ±*SD* was 3.3 ± 2.3 errors. In contrast, most participants did not exhibit omission errors, so this had a smaller distribution; nonetheless, there was a maximum of 10 errors.

**Figure 4:**
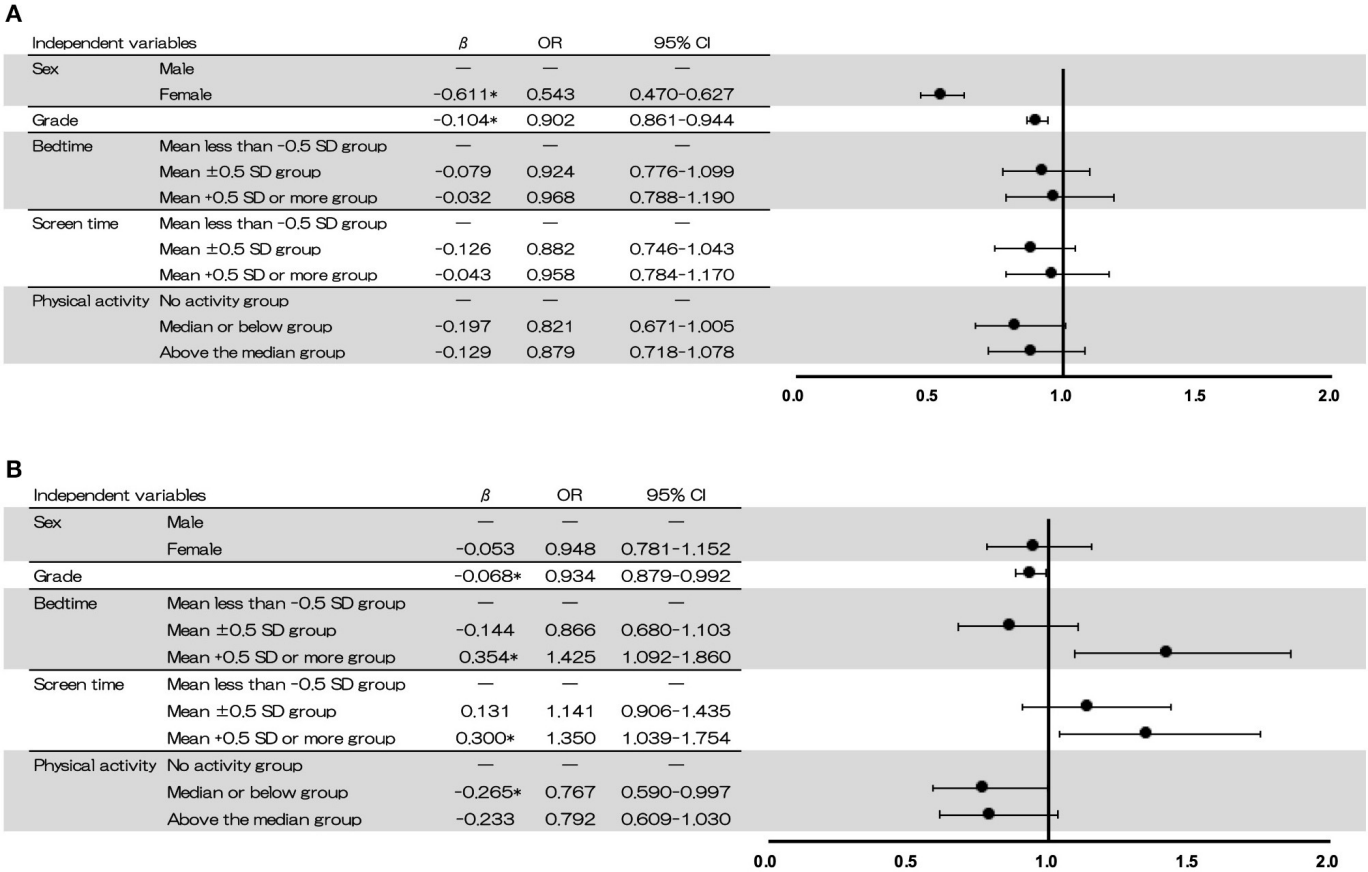
Relationship between lifestyle behaviours and commission errors **(A)** and omission errors **(B)**. The results were analysed using the data from 3rd grade of elementary school to 3rd grade of junior high school (*n* = 3,217). β = standardised partial regression coefficient, OR, odds ratio, 95% CI, 95% confidence interval.

Regarding the binomial logistic regression, for the commission errors, the variables with a significant coefficient were sex [odds ratio (OR) = 0.543, 95% confidence interval (CI) = 0.470–0.627] and grade (OR = 0.902, 95%CI = 0.861–0.944). For the omission errors, the variables with significant coefficients were grade (OR = 0.934, 95%CI = 0.879–0.992), bedtime (mean +0.5 *SD* or more group: OR = 1.425, 95%CI = 1.092–1.860), screen time (mean +0.5 *SD* or more group: OR = 1.350, 95%CI = 1.039–1.754), and PA (median or below group: OR = 0.767, 95%CI = 0.590–0.997; Figure 4).

## Discussion

### Characteristics of participants' lifestyle habits

Olds et al. (22) analysed the sleep habits of Australian children and adolescents and reported that their weekday sleep durations were similar to those of Canadian, French, and Swiss children, although slightly longer than those of children in other European countries and significantly longer compared to American children. In comparison to these results, we found that Japanese children have extremely short sleep durations. However, our results were consistent with those found in a survey conducted on Japanese children by the Committee for Surveillance of Health in School Children and Adolescents (18), and which highlight a general trend among Japanese children. Additionally, compared to the survey results mentioned above, screen time for elementary school students in our sample was 2–23 min longer and 50–62 min shorter for junior high students. Thus, our results demonstrated that our sample reported sleeping habits characteristic of Japanese children, such as shorter sleep durations, but that their screen time tended to be slightly longer among elementary school students and shorter in junior high students.

### Differences in go/no-go task performance by sex and age

Our results also indicated that, although a sex difference was observed in the commission errors, this was not the case regarding the omission errors. In contrast to our findings, Brocki and Bohlin (23) conducted multiple executive function tasks (including go/ commission tasks) on 6- to 13-year-olds and found no sex difference in terms of the disinhibition factor (which includes commission errors), but that it was detectable in terms of the speed/arousal factor (which includes omission errors). Generally, among children, commission errors in a continuous performance test reflect impulsivity, while omission errors reflect signs of carelessness (24). Based on the researchers' empirical experiences, we consider that Japanese girls tend to be more cautious than their male counterparts. Supporting this observation, Hagekull and Bohlin (25) examined the relationship between preschool temperament, environmental factors, and school-age personality in children aged 8–9 years and found that girls were more careful than boys.

Nonetheless, we wished to go further in terms of the theoretical assumptions regarding these errors to better understand the difference in the results between our study and that of Brocki and Bohlin (23). Pavlov (26) explains higher brain functions (i.e., the manifestations of the function of the human cerebral neocortex) in relation to three characteristics: 1. degree of intensity, 2. equilibrium, and 3. lability in the two neural processes (i.e., excitation and inhibition processes). He further states that it is possible to classify higher brain functions into different types when considering these characteristics. Based on this theory, Luria (27) devised a conditioned-reflexes method of grasping motion that is preceded by language instruction, namely, go/no-go tasks. Based on this understanding, we considered that omission errors may have occurred not only because the excitation process was weak (i.e., the participant made an oversight owing to carelessness) but also because the inhibition process was stronger than the excitation process (i.e., when the grasp action was suppressed by inhibitory dominance). Therefore, omission errors can reflect carelessness, inhibitory dominance, or both. As described in section 2.3.1 of this study, in view of various circumstances, our go/no-go tasks had a smaller number of trials than that conducted in many previous studies. Thus, we speculate that our results reflecting participants' impulsiveness in commission errors were not due to their carelessness identified in the omission errors; rather, we believe that the inhibition process may have been more significant in this specific condition. Therefore, the differences between our study and that of Brocki and Bohlin (23), which used 100 trials, regarding results by sex may be explained by the difference in the number of trials. In any case, further investigation is recommended to assess the relationship between the number of trials and the strength of the excitation and inhibition processes.

Our results also showed a significant difference by grade in both commission errors and omission errors. Corroborating this, van der Meere and Stemerdink (28) conducted go/no-go tests with male students aged 7–12 years and reported that those aged 7–8 years made more commission errors than those aged 9–10 years and 11–12 years. Additionally, Iida et al. (29) conducted go/no-go tasks with male and female students aged 6–12 years and reported that there was a significant negative correlation between age and commission errors under the 80% choice reaction condition in which the go task was 80% and the no-go task was 20%. Brocki and Bohlin (23) also reported that the main effect of grade was significant, and that disinhibition (comprising commission errors) and speed/arousal factors (comprising omission errors) develop with age. Specifically, disinhibition was significantly lower in children aged 9.6–13 years than in those aged 6–9.5 years, and there was a significant tendency towards speed/arousal to rapidly develop among children aged 6–7.5 years through to 7.6–9.5 years. Therefore, our findings accord with all of these findings.

### Relationship between go/no-go task performances and lifestyle habits

Our results showed that commission errors were significantly related to sex and grade, and that omission errors were significantly related to grade, bedtime, screen time, and PA. As noted, many studies have reported a relationship between sleep conditions, PA, and cognitive function using univariate analysis. This study is significant in that it analysed the relationship between multiple lifestyle habits and cognitive functions using multivariate analysis, which is likely to enhance understanding of these phenomena and their associations.

Moreover, in relation to omission errors as noted, the inhibition process may be stronger in omission errors when the number of trials is fewer, that is, lifestyle habits may have had a greater impact on the degree of inhibition compared to the impact of impulsiveness and carelessness in this study.

Previous studies have indicated that Japanese children's general lifestyle habits are concerning, as not only do they have short sleeping durations and extensive screen times but also pervasive sedentary behaviour (30, 31). These habits may further enhance the strength of the inhibition process beyond the characteristics of temperament. Correlatively, in Japan, the inhibitory type (in which the number of omission errors was higher than the standard) was found to be 0% in a 1969 survey but had increased by a few percentage points in a 1998 survey and was reported to be approximately 10% in a 2008 survey (32). The fact that this type of error was clearly observable in only a small number of children, and that omission errors are strongly related to lifestyle habits, which has been a cause for concern in recent years, highlights an issue that requires further investigation. The mechanism of these errors is not yet known, and clarification on this point is needed urgently.

### Limitations and recommendations for future research

Although our study presents relevant additional insight to the literature, it also has limitations that need to be considered in future studies. First, as our study was cross-sectional, it could not identify causal relationships; thus, future longitudinal studies are warranted. Second, we utilised a survey to collect data on children's lifestyle habits. Future studies should collect objective data on lifestyle habits while conducting an investigation similar to ours to allow for between-study comparisons. Third, whether each omission error resulted from participants' carelessness/inattention or inhibitory dominance remains to be examined; thus, future studies are warranted to investigate the underlying mechanisms of omission errors using other indicators (e.g., simple and choice reaction time).

## Conclusions

We found that children's cognitive functions are related to their lifestyle habits (i.e., sleep conditions, screen time, and PA) in addition to sex and grade. Our results may provide important guidance in reforming daycare and educational practises to address the changing higher brain function profiles of contemporary children.

## Data availability statement

The original contributions presented in the study are included in the article/supplementary files, further inquiries can be directed to the corresponding author.

## Ethics statement

The studies involving human participants were reviewed and approved by Ethics Review Committee for experiments on humans, Nippon Sport Science University. Written informed consent to participate in this study was provided by the participants' legal guardian/next of kin.

## Author contributions

AS and SN contributed to conception and design of this study and interpretation of data. AS conducted the acquisition of data and contributed substantially to the analysis. AS drafted the manuscript and SN had approved the final manuscript. All authors contributed to the article and approved the submitted version.

## Funding

This work was supported by JSPS KAKENHI, Grant Number JP18K13125.

## Conflict of interest

The authors declare that the research was conducted in the absence of any commercial or financial relationships that could be construed as a potential conflict of interest.

## Publisher's note

All claims expressed in this article are solely those of the authors and do not necessarily represent those of their affiliated organizations, or those of the publisher, the editors and the reviewers. Any product that may be evaluated in this article, or claim that may be made by its manufacturer, is not guaranteed or endorsed by the publisher.
